# Tracking Post-Hibernation Behavior and Early Migration Does Not Reveal the Expected Sex-Differences in a “Female-Migrating” Bat

**DOI:** 10.1371/journal.pone.0114810

**Published:** 2014-12-17

**Authors:** Dina K. N. Dechmann, Martin Wikelski, Katarina Varga, Elisabeth Yohannes, Wolfgang Fiedler, Kamran Safi, Wolf-Dieter Burkhard, M. Teague O'Mara

**Affiliations:** 1 Max Planck Institute for Ornithology, Department of Migration and Immuno-Ecology, Am Obstberg 1, 78315 Radolfzell, Germany; 2 University of Konstanz, Department of Biology, 78457 Konstanz, Germany; 3 Smithsonian Tropical Research Institute, Balboa, Ancón, Panama; 4 Gumpisloch 2, 8597 Landschlacht, Switzerland; University of Western Ontario, Canada

## Abstract

Long-distance migration is a rare phenomenon in European bats. Genetic analyses and banding studies show that females can cover distances of up to 1,600 km, whereas males are sedentary or migrate only short distances. The onset of this sex-biased migration is supposed to occur shortly after rousing from hibernation and when the females are already pregnant. We therefore predicted that the sexes are exposed to different energetic pressures in early spring, and this should be reflected in their behavior and physiology. We investigated this in one of the three Central European long-distance migrants, the common noctule (*Nyctalus noctula*) in Southern Germany recording the first individual partial migration tracks of this species. In contrast to our predictions, we found no difference between male and female home range size, activity, habitat use or diet. Males and females emerged from hibernation in similar body condition and mass increase rate was the same in males and females. We followed the first migration steps, up to 475 km, of radio-tagged individuals from an airplane. All females, as well as some of the males, migrated away from the wintering area in the same northeasterly direction. Sex differences in long-distance migratory behavior were confirmed through stable isotope analysis of hair, which showed greater variation in females than in males. We hypothesize that both sexes faced similarly good conditions after hibernation and fattened at maximum rates, thus showing no differences in their local behavior. Interesting results that warrant further investigation are the better initial condition of the females and the highly consistent direction of the first migratory step in this population as summering habitats of the common noctule occur at a broad range in Northern Europe. Only research focused on individual strategies will allow us to fully understand the migratory behavior of European bats.

## Introduction

Strong seasonal variations in climate and resource availability impose increased energetic pressures on animals. This may then enhance sex differences in behavior and ecology, particularly in strongly energetically limited species and species with a high degree of specialization [1]. Females and males of a single species may use varying strategies to cope with seasonal environmental fluctuations, which can be expressed in differences in behavior, physiology, morphology as well as ecology often resulting in sexual segregation [2], [3]. These differences can be so large that the sexes could be treated as separate ecological species or conservation units (e.g. [4]–[6]).

In temperate bat species where sexual segregation is common [7]–[9], sexual size dimorphism is absent or female biased by only a few percent or less [10] and thus allometry is an unlikely driver of sexual segregation. Most temperate bat species mate in the fall, and this is often the only time the sexes meet. Once they rouse from hibernation in the spring, females start a race against time to use the short, but resource rich warm season to gestate offspring that can weigh up to 1/3 of maternal body weight at birth [11], and nurse offspring until they reach independence at nearly 90% of adult size. During this time females should reduce the energy saving strategy of daily torpor, which is frequently used by males and non-reproductive females as it interferes with the development of the embryo and milk production [12]–[15]. In addition to this extreme reproductive burden, females must also store energy before the next winter while leaving themselves enough time to find suitable mating partners. In non-migrating parti-coloured bats (*Vespertilio murinus*) large differences between the sexes in activity, habitat selection, migration distance and home range size have been reported [6], [16] and are likely a result of this high energetic pressure on females.

In most migratory species, including bats and other mammals, both sexes migrate. However, in temperate zone bat species migration can be female biased, resulting in an extreme form of sexual segregation where females migrate to natal maternal colonies and males usually remain near over-wintering sites or move relatively shorter distances [17]. The common noctule (*Nyctalus noctula*) is one of the few European bat species that twins [18], [19], and only females migrate long distances [20], [21]. This extreme reproductive and energetic cycle, amplified by the need to dedicate time and foraging effort to increase body condition prior to migration should enhance differences in physiology and behaviors between the sexes in this species. Each year, females from migratory populations fly north or north-east, covering distances of up to 1600 km from the wintering habitats (reviewed in [21]) to return to their natal colony. Males, in contrast, are thought to perform only one long-distance migration when they leave the natal colony and disperse to a mating site. Males are then presumably more or less sedentary, moving only smaller distances of up to 100 km [21] and mate with females from different populations as they move through male habitats during fall migration [20], [22].

Physiological preparations are needed for long-distance migration. In birds this has been well studied (e.g. [23], [24]) and the accumulating evidence in bats indicates similar convergent physiological, biochemical and energetic changes prior to migration [25]; reviewed in [26]. However, two significant differences between bird and bat migration are noted. First, and predominantly, most bat females are pregnant during the onset of migration, while in birds migration (by both sexes) is temporally distinct from reproduction. Second, many songbird species of sizes similar to bats migrate at night when they are typically not active. This leaves them much of their typical active period to forage and refuel; however, they may expend more energy during this time than over the actual migratory periods [27]–[29]. Bats also migrate at night, during their usual foraging period. This results in a potential trade-off between migration and foraging and a need to build the fat reserves that, once bats have metabolized food ingested during foraging, fuel migration [30]. This temporal and energetic restriction should enhance physiological differences between the sexes in the post-hibernation period.

The high cost of reproduction in female common noctules is reflected in differences between reproductive and non-reproductive individuals in their behavior and habitat selection [6], [31]. This suggests that reproductive females undergo high energetic output and that pre-migration body condition preparation may be crucial for successful reproduction. Sex-differences in noctule foraging behavior, diet, and changes in body condition, particularly during the spring pre-migration period should therefore be at least as pronounced if not more so than in a sedentary species. To date there are limited data during the spring pre-migratory period in temperate bats as research has largely focused on the locally accessible, conservation relevant and conspicuous maternity colonies. However, new and rediscovered technical approaches now allow more in depth research on these cryptic and elusive animals.

We compared early spring body condition, foraging behavior, and migration timing of female and male noctules using a variety of methods. In particular we predicted that 1) females emerge from hibernation with similar or better [32] body condition compared to males, and gain mass more rapidly, 2) females use larger home ranges, have longer and/or more foraging sessions and/or select different foraging habitat, 3) once prepared for migration, only females will leave the local area and migrate away in a directional manner and 4) these differences should be reflected in the isotopic signatures of feces (local foraging) and hair (formed after molting during the summer).

Confirming or rejecting these predictions will help us to deepen our understanding of the evolution of sex-biased behavior in mammals, particularly sex-biased migration, under different energetic pressures. Such knowledge will contribute to efficient conservation strategies, but also will help to describe the overlap of the niches the two sexes in this and potentially other species may occupy.

## Methods

For morphological data we captured 282 adult common noctules (*Nyctalus noctula*) during April-May of 2012–2014 from three sites in Switzerland (Seeburgpark Kreuzlingen: 47.649928°, 9.186123°; Allmend Frauenfeld: 47.580268°, 8.906434°; Bischofszell: 47.485279°, 9.218046°) where bats regularly use boxes during winter hibernation and post-hibernation when preparing for migration. Allmend Frauenfeld and Bischofszell are 20–25 km southwest and south, respectively, of the main study site at the Seeburgpark Kreuzlingen. Boxes were checked two to three times between April and May, and all measurements (see below) were taken from all bats. Bats were also captured during evening emergence from a migratory stopover site in the roof of a school near Konstanz, Germany (Reichenau-Waldsiedlung: 47.696738°, 9.117721°).

Upon capture or removal from the box, bats were individually placed in soft cloth bags until processing According to a standard protocol, each bat was, weighed (±0.5 g with a Pesola spring balance), the forearm measured (±0.1 mm with calipers), and the sex and reproductive status (nulliparous/postlactating for females; reproductively active or not as assessed by the filling of the epididymes for males) determined. We took a cutting from the dorsal hair and, if provided, a fecal sample (for stable isotope analysis). Finally, each bat was marked with a subcutaneous pit-tag (ID100; Euro ID, Weilerswist, Germany), injected under the dorsal skin. A subset of the bats in 2012 and 2013 were then equipped with a radio-transmitter (see below and S1 Data). We released all bats within an hour either at the site of capture or placed them in the box where we had found them.

For all analyses of body condition, radio tracking, and stable isotope we present results as means ± sd.

### Ethics statement

All handling and sampling of the bats in Switzerland was approved by the Veterinäramt Thurgau (permit nr. FIBL1/12), and work in Germany was approved by Regierungspräsidium Freiburg (35-9185_81/G-12/16). All methods conformed to the ASAB/ABS Guidelines for the Use of Animals in Research.

### Body condition changes

To determine whether females gain weight more rapidly than males in preparation for the (longer) migration we captured bats early in the spring, presumably soon after rousing from hibernation (mid-April) and just before migration (early May). The mass and forearm measurements were then used to calculate body condition (mass divided by forearm length) to account for the larger relative size of the females [21]. Using the *lme4* package in R 3.0.2 we generated generalized linear mixed effects models (GLMM) that incorporated capture year and capture site as random effects to compare sexes. To compare a single sex across sites, we used year as a random effect. Fixed effects were evaluated by comparing nested models that differed in the factor of interest (e.g., sex) with a likelihood ratio test (χ^2^) [33].

### Radio-telemetry – local foraging

Bats were tracked in 2012 and 2013 with complementary methods. For the observation of foraging behavior, home range size, activity and comparison of habitat use we equipped bats with external radio-transmitters (BD2, Holohil, Canada, see below). Bats were then released as above and radio telemetry started the following night. We used three models of wide range telemetry receivers (AR8000/8200, AOR Ltd; Sika, Biotrack) in combination with collapsible H- or Yagi-antennas. Between two to four tracking teams of two persons each were placed at elevated points around the bats' roosts. Tracking teams scanned through the frequencies of the between two to nine simultaneously tagged bats at predetermined intervals (1–2 minute scanning interval depending on the number of tracked bats, i.e., each bat was triangulated at least three times per hour) and determined the direction from which the strongest signal was received as well as whether the bat seemed to be moving or not (as assessable by the variability in signal strength). From the position of the tracking team, the direction of the signal and the intersection of the resulting lines the position of the bats was then triangulated. We used homing in as well as scans from an airplane to find stationary bats during the day.

In 2012 bats for radio-tracking were caught when emerging at dusk from the roof at Reichenau-Waldsiedlung and from roost boxes in the surrounding forest. Ten females (27.9±2.5 g) and 6 males (27.4±2.6 g) were fitted with 0.5 g Holohil Lb-2 radio transmitters. Two transmitters were attached using a silicone-based skin glue (Sauer Hautkleber, Manfred Sauer, Germany) directly to clipped fur between the scapulae. Because one of the bats removed the transmitter in the first night, the remaining 14 transmitters were attached with superglue. Transmitters weighed 1.82±0.17% of the bat's body mass, and were well within the recommended 5% range [34]. Nine individuals dropped their glued transmitter after only 2±2 days and seven animals migrated immediately after transmitter attachment, as they were not located during daily searches via airplane-mounted antennae and receivers. Bats were captured on April 24 (2 bats), April 27 (2 bats), and April 30 (12 bats) and were tracked beginning the night after capture for a total of 12 nights. Bats were tracked from shortly before sunset until 6am and locations for each bat were estimated every 15 minutes. We also recorded the beginning and end time of each bats' foraging sessions to determine differences in activity.

In 2013 bats were removed during the day from roost boxes in the Seeburgpark. Eight females (26.9±2.9 g) and 10 males (25.7±2.9 g) were fitted with 0.85 g Holohil LB-2 radio transmitters using a collar with a degradable weak link (O'Mara et al. 2014). Transmitters were sewn to a 3 mm shoelace that was then fitted to the bat's neck and secured in place with degradable braided glycolic acid suture (Safil-C, Braun, Aesculap). Transmitters and collars were 0.92 g and weighed on average 3.9±0.5% of the bat's body mass. Bats were tracked 23±17 days, the collars extending tracking through transmitter battery life.

Bat locations were estimated using handheld radio receivers and antennae from 2–3 locations in the Konstanz area, depending on the location of the bats until April 28. From April 28-May 14 bats were tracked every second night, and until June 10 were monitored for presence or absence. Data collection began the day following transmitter attachment (April 22: 7 bats, April 24: 8 bats, April 29: 1 bat) and lasted from just before sunset until 3 or 4 am to capture several foraging sessions. Again, we used an airplane-mounted antennae system to locate animals that were not within radio contact during foraging sessions.

All analyses were conducted in R 3.0.2 Individual locations were estimated using triangulation data in *geosphere*. These locations were then imported to a *move* object where 95% minimum convex polygons (MCP) were calculated in *adehabitatHR*. Triangulated locations for all bats are stored on Movebank (movebank.org) and are accessible at doi: 10.5441/001/1.f01815nq.

### Radio-tracking - migration

In the mornings one of us (M. Wikelski) searched for the bats' roost in an airplane (Cessna 172; [35] using the same tracking material as the ground tracking teams to determine which bats had left for migration, their migration direction, as well as the distance to the next stop-over. Locations were then plotted to illustrate migration direction and distances by male and female bats. Migration data are stored on Movebank (movebank.org) and and are accessible at doi: 10.5441/001/1.f01815nq.

### Stable isotope analysis

Stable isotopes are reliable indicators of diet [36] and can be used to trace the sources of organic matter to terrestrial or aquatic [37]–[40]. In general, predatory and animals from freshwater systems have higher stable nitrogen isotope (δ^15^N) and more negative stable carbon isotope (δ^13^C) values than herbivores or terrestrial animals. Isotopic ratios of sulphur (δ^34^S) are also useful for discriminating origins from terrestrial versus marine systems. Moreover, stable isotope ratios vary geographically and can help identify residency and movement patterns in slower-growing and inert, keratin tissues such as hair and fur [41], [42]. Hair has been used to indicate the residency and habitat selection patterns in several bats species [37], [43], [44] and to give evidence for summer molting of fur and female biased long-distance migration [45]. Noctule hair is generally molted once per year and regrown in June/July by males and in July/August by females [18], [21]. Noctule hair then is composed of dietary input of bats during this time and can reflect variation in foraging habitat (aquatic vs. terrestrial) as well as variation in soil and geological types.

We used a triple isotope approach (δ^13^C, δ^15^N and δ^34^S) to identify variation in diet and summer residency using feces and hair, respectively. We collected feces and hair samples for stable isotope analysis during all captures in spring 2012 and 2013. Feces, if available, were collected from the cloth bags the bats were individually placed in while waiting to be processed. Fecal samples were then stored at −20°C and hair samples at room temperature until further analysis. For analysis the fecal samples were homogenized with a spatula, placed in a drying oven (50°C) for 16 hours and homogenized again with a mortar and pestle.

Hair clippings were taken from the back of each bat. Hair samples were washed with pure ethanol and then also placed in the drying oven for three days. Samples were then weighed (±0.001 mg) and 1–1.2 mg (hair) or 0.8–1.0 mg (feces) placed in a tin capsule. An elemental analyser interfaced to a continuous flow isotope ratio mass spectrometer was used. Several replicates of standards (calibrated against IAEA, Vienna, reference materials) were run to obtain a samples ratio relative to reference gases.

Isotope values were analyzed using multivariate analysis of variance to identify if there were significant sex differences in the mean values derived from our sample. We were also interested in differences in the amount of variation in females and males, as this may help identify if there were sex differences in the dietary breadth or in the summer geographic origin among the individuals of each sex. To test this we calculated the coefficient of variation (CV) for each isotope type per sex and used a bootstrap approach [46] to generate a null distribution of pooled samples. We could then identify if each sex's CV was within the 95% confidence interval of this bootstrapped sample.

## Results

### Body condition changes

**Figure 1:**
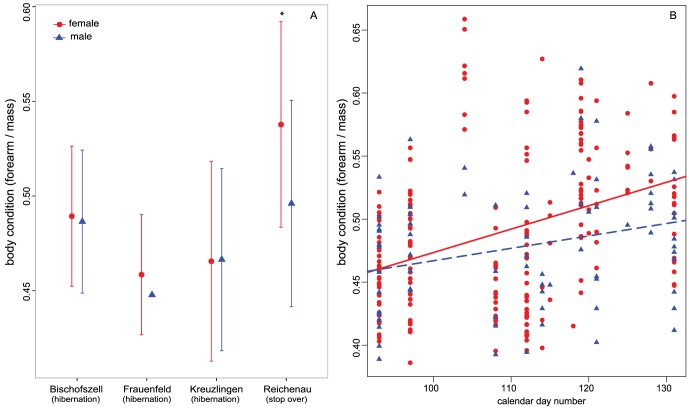
Body condition (mean ± sd) for female and male noctules at the A) hibernation (Bischofzell, Allmend Frauenfeld and Seeburgpark Kreuzlingen) and migration stopover sites (Reichenau-Waldsiedlung) four capture sites. The asterisk [*] indicates that females captured at the stopover site have significantly higher body condition than females at all other sites. B) Body condition increases with day number for both sexes, but there are no significant differences between females and males. Lines for males and females are only for illustration purposes.

In general, noctule females were larger and heavier than males. Females had longer forearms (females: 54.40±1.28 mm; males: 53.65±1.78 mm; t = 3.209 df = 95, p-value  = 0.001), and were on average ∼1 g heavier than males around the time of emergence from hibernation (females; 26.69±3.36 g; males 25.52±2.87; t = 2.928, df = 162, p = 0.003). However, females are not in better condition when we scaled body mass by forearm length, and when we included site and year as random effects, females did not have higher body condition than males early in the spring (female: 0.49±0.06, male: 0.48±0.05;χ^2^ = 3.763, df = 1, p = 0.052; Fig. 1A). Females that were captured at the migration stopover site (Reichenau), however, had higher body condition than females captured at the hibernation sites (i.e. roost boxes, χ^2^ = 69.12, df = 3, p<0.001; Fig. 1A), but there were no site differences in body condition in males (χ^2^ = 6.251, df = 3, p = 0.10). Body condition in both sexes increased with day number (Fig. 1B); however there was no significant difference between females and males in the rate of increase (ANCOVA F = 0.655, df = 1, p = 0.414).

### Radio-telemetry – local foraging

**Figure 2:**
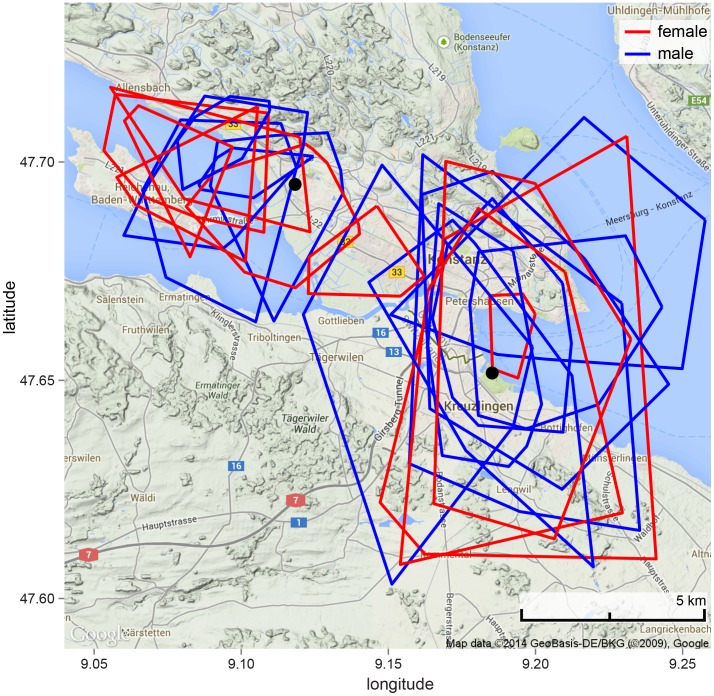
Noctule 95% minimum convex polygons calculated from triangulated locations. Females and males used the same foraging locations and the same range size. Black dots indicate the capture sites.

We calculated 95% MCP for 23 individuals (10 females, 13 males) across the two tracking years. From a total of 158 bat-nights of tracking (5.6±3.4 nights per bat, 3.8±3.0 locations per night) we did not find any sex differences in the size of home ranges of bats in the local foraging areas (t = 0.262, df = 15, p-value  = 0.797; females 2051±2096 m^2^, males: 2252±1391 m^2^) or in the habitats used (Fig. 2) during both years. Males and females also used the same number of foraging bouts per night (female: 1.65±0.80 bouts; male: 1.61±0.64 bouts; t = 0.36, df = 224, p = 0.72) for the same amount of total time per night (female: 54.0±56.4 min; male: 42.9±45.7 min; t = 1.22, df = 116, p = 0.23). However, females show a higher maximum number of foraging bouts than males (6 vs. 3, respectively), and a longer maximum amount of time foraging per night (278 vs. 180 min, respectively). Both males and females predominantly used small home ranges over and near Lake Constance.

### Radio-tracking - migration

**Figure 3:**
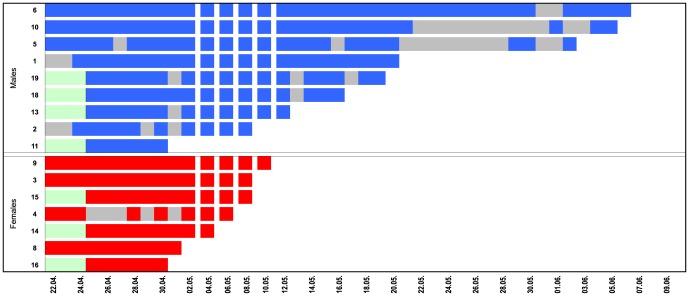
Duration of residency during the 2013 tracking season. All females (bottom, red) left the study area, and several males (top, blue) left the study area for periods of several days or permanently. Grey bars indicate days when bats were not found in the local foraging area even though some were located by airplane. Local dispersal of males towards end of season not included in the figure.

**Figure 4:**
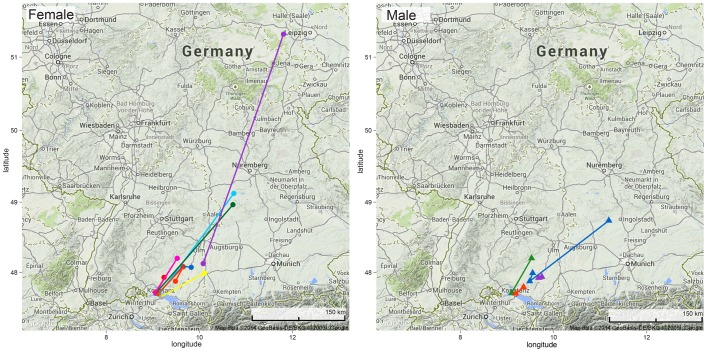
First migration steps in female (left) and male (right) noctules.

Females remained in the local foraging area for a maximum of 14 days before migrating (Fig. 3). In 2012, several animals disappeared from the study site before we were able to track them from the airplane. Across the two study years, we were able to track the first migration steps of 8 females and 3 males (Table 1). Surprisingly, both males and females were found to travel significant distances from the local foraging area all in the same general northeasterly direction (Fig. 4). However, all females, but only some of the males migrated and some of the latter later returned to the study area and then dispersed only locally (data not shown). The maximum distance we observed was 475 km for a female and 208 km for a male. One female was found the next day after leaving the lake Constance area. She had travelled a distance of 180 km. When she is excluded from the data, female mean travel rate was 24.3±22.2 km per day (Table 1). Males travelled on average 14.2±5.6 km/day. However, all other individuals were located only several days after leaving the study area and thus this single female's data point indicates the migration speeds that are possible by this species.

**Table 1:** Distances traveled during migration steps.

Year	Sex	Distance traveled (km)	Number of days	Average migration rate (km/d)	Number of fixes	Maximum speed along route (km/d)
2012	Female	200	3	66.7	2	131
	Female	180	1	180.0	1	180
	Female	80	12	6.7	2	6.7
	Male	70	8	8.8	2	11.0
2013	Female	43	4	10.8	1	10.8
	Female	52	2	26.0	1	26
	Female	475	12	39.6	2	43.1
	Female	80	6	13.3	1	13.3
	Female	60	9	6.7	2	7.0
	Male	20	1	20.0	1	20.0
	Male	208	15	13.9	2	17.3

### Stable isotope analysis

#### Diet

**Figure 5:**
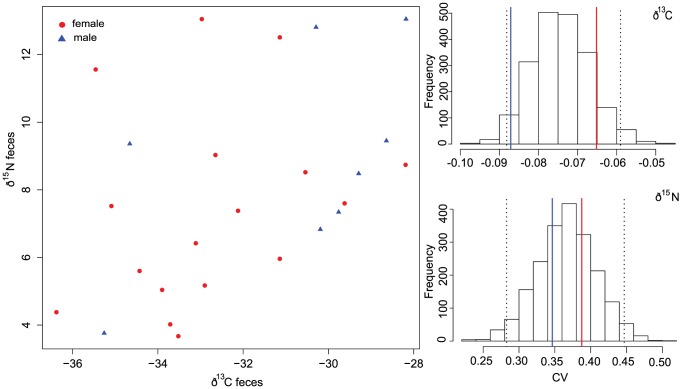
Feces stable isotope values (δ^13^C and δ^15^N) for females (red circles) and males (blue triangles). A) Values of δ^13^C vs. δ^15^N extracted from feces and B) Bootstrapped values of the coefficient of variation for both males and females combined. Vertical dashed lines denote the 95% confidence interval and solid lines show female (red) and male (blue) CV.

Stable isotope ratios from feces directly reflect a bat's ingested diet. In general, there were no sex differences in mean fecal isotope values of the sampled 17 females and 8 males for δ^13^C (females: −32.75±2.13; males: −30.78±2.7) δ^15^N (females: 7.42±2.87, males: 8.89±3.07) or δ^34^S (females: 0.85±1.11; males: 0.6±0.7), and we detected no sex differences in distribution of the combination of all three isotopic values (MANOVA Wilks lambda  = 0.8, F_1,23_ = 1.7, p = 0.20). There were no sex differences in the coefficient of variation of δ^13^C, δ^15^N, or δ^34^S (Fig. 5 and S1 Figure and S2 Figure as well as S1 Data).

#### Residency

**Figure 6:**
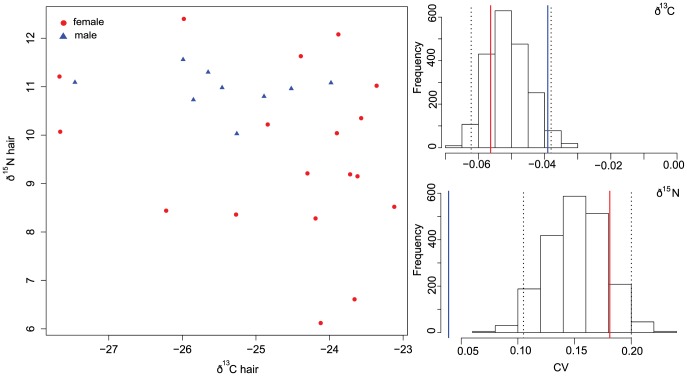
Hair stable isotope values (δ^13^C and δ^15^N) for females (red circles) and males (blue triangles). A) Values of ð^13^C vs. ð^15^N extracted from hair and B) Bootstrapped values of the coefficient of variation for both males and females combined. Vertical dashed lines denote the 95% confidence interval and solid lines show female (red) and male (blue) CV.

Noctule hair is regrown during the summer prior to fall migration and therefore reflects the isotopic signature of the summering habitat. Similar to the fecal analyses, we did not find sex differences in the mean values in the hair isotopic ratios of 18 females and 9 males for δ^13^C (females: −24.63±1.38; males: −25.45±0.99), δ^15^N (females: 9.6±1.73 males: 10.94±0.42) or δ^34^S (females: 2.05±2.8; males: 2.02±1.11). We also did not detect sex differences in distribution of the combination of all three isotopic values (MANOVA Wilks lambda  = 0.8, F_1,25_ = 1.9, p = 0.15). However, there were significant sex differences in the CVs, for both δ^15^N content and δ^34^S, with males showing lower levels of variation in the isotopic ratios of these two elements than females (Fig. 6 and S1 Figure and S2 Figure), likely indicating that the females sampled were sourced from a broader geographic range than the males, and that these females differed in their use of terrestrial and aquatic feeding habitats. There was no sex difference in the CVs of δ^13^C.

## Discussion

Sex differences in behavior and ecological niche (“ecological dimorphism” [47] have been found in many species and can be so profound as to be of large conservation relevance [48], [49]. The combination of sex-biased migration and high energetic and reproductive pressures on female noctules led us to predict large behavioral sex differences immediately after emergence from hibernation and during preparation for spring migration. However, while all differences we found pointed in the expected direction almost none of them were significant. We confirmed slightly larger size of females [21] and found corresponding significant sex differences in body condition, as well as higher body condition scores in migrating females (from the stop-over roost) compared to females that had not started migration yet. Different migratory behavior was also confirmed by larger variation of stable isotope signatures in female hair. However, there were no sex differences in body condition increase over time, in the foraging behavior, or in dietary composition estimated through stable isotope analysis. Surprisingly, we found that both males and females moved significant distances from the capture area - all in the same northeasterly direction. While some males remained resident and/or returned after short absences, all radio-tracked females migrated away from the study area.

One important requirement for migration is physiological preparation for the high energy expenditure enhanced by a compromise between time spent migrating, foraging and resting [27], [28]. All evidence accumulated to indicates this is true for migratory bats as well [26], [50]. Because fat is the primary fuel for migratory flight in bats [25], [30], [51], [52], migratory preparation should be reflected by mass gain. While our females rouse from hibernation in better condition than the males, similar to what has been shown in *Myotis lucifugus* [32] they do not gain mass more quickly than the males right away. However, both sexes gain mass quite rapidly during this time. For example in Kreuzlingen in 2014 females increased mean mass by 5.6 g from 24.8 g (n = 35) to 30.4 g (n = 7) in the 18 days from first to last capture and males from 24.4 g (n = 18) to 28 g (n = 2). Females caught from a larger stop-over roost containing individuals that had either started migrating from further south or aggregated there from hibernacula were caught 8 days before the second capture in Kreuzlingen with a mean mass of 33.4 g (n = 7), much heavier than the local females until departure. Males from the stopover roost weighed 28.5 g (n = 2) the same as in Kreuzlingen eight days later. This seems to indicate that female preparation for migration may not be completed at the hibernation site, but partway through migration. This is confirmed by our radio tracking data that showed that females were found stationary at relatively short distances from the hibernation site up to 12 days after leaving our study area before disappearing. It is also possible that both sexes may simply not be confronted with a resource bottleneck in early spring at our study site (a productive lake environment), both gaining mass at maximum rates and the females continuing to do so after the onset of migration.

As previously reported we found a slight but significant larger size of the females, but no difference in body condition in the over all dataset. However, the heaver mass of the females in the stopover roost, i.e. during migration hints that sex dimorphic behavior may be linked to morphological differences. More detailed work is needed to interpret the role of the size dimorphism, i.e. whether this is an absolute size difference according to the “big mother hypothesis” [53] or an adaption to other differences between the sexes, such as migration distance (O'Mara/Dechmann et al. in prep) or ecological niche [47]. It is possible that males save mass increases because they do not have to migrate (as far) and prefer to remain relatively lighter to maintain lower wing load. Alternatively, bats may need to hold lower overall fat stores, as fat turnover is quite high and may necessitate multiple stopovers for refueling [25], [30]. Birds can have very different refueling strategies depending on the species, with some making more or less extended stopovers for refueling and others accumulating all the energy they need prior to migration and then covering the distance much more rapidly [28]. More work on the location, duration and quality of stopovers will be necessary along with monitoring the bats' physiology to answer these questions. The noctule is hypothesized as partial migrant with resident and migrating individuals or even populations [21] based on disappearance of the females from the wintering range in the spring and their reappearance in the autumn. Comparisons with populations from further north that are more sedentary or migrate shorter distances would help identify these differences in migration strategy.

The interpretation that resources may not be limited which allows both sexes a maximum initial mass increase after hibernation is supported by our local tracking data. In other species with a similar ecological niche males use lesser quality habitat to forage, presumably to avoid competition with females during the reproductive season in the summer [6], [9], [16]. Not only did we not find a sex difference in habitat use or home range size, but also home range size was overall very small. In fact, stable isotope analyses confirm that both sexes fed on the same resources during our study. Resource availability may allow them to forage in the same area without competition and with minimal energetic investment. Comparative tracking data from noctules at other sites are only available from the summer, after spring and before fall migration where home ranges of both reproductive and non-reproductive females were about four times larger than in our study [31]. Other species with a similar ecological niche, in particular the smaller congeneric Leisler's bat (*Nyctalus leisleri*), also had larger home ranges during the pre-migration period in the spring [54], [55]. One important difference between our study and previous work may be the study area: our bats were all caught within a maximum distance of a few hundred meters from the lakeshore and were likely in a more productive habitat than in the mosaic English habitat [31]. Noctules are narrow-winged aerial hawkers that depend to a large extent on swarming insects as a resource [56]–[58]. Gloor and co-authors [57] showed that noctules from a Swiss population in April and early May consumed a high percentage of chironomids – a dipteran family with aquatic larvae that typically emerge in simultaneous masses from water surfaces. Tracking female noctules in a habitat without large water bodies showed significant differences between lactating and non-reproductive females that are likely related to elevated costs of reproduction in lower quality environments [31].

The most surprising result from our tracking data was the documentation of both male and female migration. While our isotope data indicate a difference in the long-distance migration behavior, at least initially both sexes can migrate. However, only a subset of the males, but all females left the study area and several of the males that left returned to the study area later during the study period. The general sex-biased pattern of female migration to natal maternity colonies and male residency in other parts of the range is well documented from recaptures [21] as well as confirmed by population genetics [22]. While one could have expected the males to disperse from the wintering site to establish individual home ranges for the summer, our airplane surveys showed that both sexes move in the same northeasterly direction. Petit and Mayer [20], [59] suggest that males may scatter along the female migration route in preparation for the female return in August and it may be necessary for the males to establish these territories early on, perhaps with those meeting the returning females earlier in the late summer gaining a reproductive advantage. This would also partially explain the consistent direction of the movement, which is surprising as genetic evidence and surveys of maternity colonies suggest that females retain philopatry to their natal colonies, while hibernacula contain random aggregations of individuals [59]. Our recapture data confirm this. After three years of captures and confirmed hibernation in artificial roosts at the Kreuzlingen site, we recapture only 2–4 individuals (less than 5%) each autumn. An additional explanation for the consistent direction during early migration may be that migration direction, which could be anywhere from due North up to much further East based on evidence from recaptures and genetics, is so consistent because all individuals use similar landscape elements, such as rivers and wind [28]. The increasing numbers of bat casualties at wind power plants during the migratory season confirm that at times apparently narrow movement corridors exist [60], a phenomenon of increasing conservation concern.

Also interesting is the emerging information on migration speeds as it is slower than predicted flight speeds for this species. Averaged over the entire period between disappearance from the study site and rediscovery of the migrated bats most of the resulting speeds fall well below the predicted maximum range speed based on mass and wing morphology [61]. Recorded foraging speeds of noctules are 53 km/h [62] and up to 60 km/h [63] and recorded speeds from bats with similar morphology are in the same range (reviewed in [54]). However, our bats' mean travel rates fall well below that. If bats follow a pattern where they migrate in steps that are interrupted by necessary refueling stopovers, most of them may have covered the full distance to the locations where we observed them in one or just a few nights. Our observed distances between 66 and 180 km (table 1) in one night may not be unusual.

One important step forward within our study that made the observation of the long-distance migration, sometimes after quite a long time period depending on availability of the plane, possible was the use of our customized collars instead of glued-on transmitters. This new technique vastly extended the time transmitters were on bats (see also [64] for details) and allowed us to make full use of the transmitter life time of animals that stayed within range and thus to distinguish properly between animals that left the study areas, those that remained and those that left only temporarily. This technical advancement, given proper logistical resources and manpower, now allows for tracking of the full migration of the females even in an animal as small and nocturnal as the noctule for which no appropriately sized and long-lived GPS and satellite solutions are available yet.

In conclusion, we cannot confirm a strong difference in energetic pressures on male and female noctule bats in the spring even though we find evidence for differences in migration behavior. Thus, we propose an alternative potential interpretation of our results: migration in noctules may simply not be overly costly. Although this has not been shown for noctules, migratory bats can easily fly six hours during foraging (*Lasiurus cinereus*, [65] covering extremely large distances during commutes to and from the foraging areas (e.g. [6], [54], [66]) and gaining speeds of 30–40 kph or more [54], [67], [68]. In addition, if migrating bats take refueling breaks migration might not be as costly as currently assumed and may not require significant mass gain. One also needs to keep in mind that pregnancy in bats is less costly than lactation, especially early pregnancy [69], [70]. The rates at which our bats gained mass in the spring indicate that females may save weight during early migration, thus flying more cost–efficiently and then gaining the additional reserves required for reproduction later during the pregnancy.

## Supporting Information

S1 Figure **Hair (left) and feces (right) stable isotope values (δ^13^C and δ^34^S) for females (red circles) and males (blue triangles).**
(EPS)

S2 Figure **Bootstrapped values of the δ^34S^ coefficient of variation for hair (left) and feces (right).** Vertical dashed lines denote the 95% confidence interval and solid lines show female (red) and male (blue) CV.
(EPS)

S1 Data **Capture records including sex, forearm length (FAL), body mass, body condition (bc) and isotopic values of ð^13^C, ð^15^N, and δ^34^S in hair and feces from** ***Nyctalus noctula*** **.**
(CSV)
